# Effects of early- and late- neuraxial analgesia on multiparous women: a retrospective monocentric study

**DOI:** 10.1186/s12871-023-02395-4

**Published:** 2024-01-02

**Authors:** Wenxing Cheng, Chunyu Xiong, Hao Li, Jiao Wen, Jing Peng, Shiyao Wu, Haotian Pan, Lei Chen, Weina Xia, Yun Zhao

**Affiliations:** 1 Department of Obstetrics, Maternal and Child Health Hospital of Hubei Province, Tongji Medical College, Huazhong University of Science and Technology, No. 745, Wuluo Road, Hongshan District, Wuhan, 430070 China; 2 Wuhan University of Science and Technology, No. 2, Huangjiahu West Road, Hongshan District, Wuhan, 430065 China; 3 Department of Maternal and Child Health Hospital of Hubei Province, Hubei University of Medicine, Hubei University of Medicine, No. 745, Wuluo Road, Hongshan District, Wuhan, 430070 China

**Keywords:** Neuraxial analgesia, Duration of stage, Intrapartum cesarean delivery, Intrapartum fever, Multiparous women

## Abstract

**Background:**

The mechanism underlying maternal fever and prolonged labor progression associated with neuraxial analgesia (NA) remains elusive, raising concerns among certain pregnant women regarding the application of NA during vaginal delivery. This study aimed to investigate the impact of early and late NA on maternal and neonatal outcomes in multiparous women.

**Methods:**

This retrospective study collected data from 1119 multiparous women with singleton pregnancies, full term and live births at our labor and delivery center between August 1st, 2021 and July 31st, 2022. Based on the timing of NA initiation, participants were categorized into three groups: no-NA, early-NA and late-NA. The no-NA group comprised of 172 women who did not receive NA during vaginal delivery; the early-NA group included 603 women in which NA was initiated when cervical dilation was between 0.5 and 2.0 cm; and the late-NA group comprising 344 cases in which NA was initiated at the cervical dilation of ≥ 2 cm. Maternal and neonatal outcomes were observed, including durations of the first, second, third and total stage of labor, the rate of intrapartum cesarean delivery (CD), intrapartum fever, postpartum hemorrhage (PPH), transfer to intensive care unit (ICU), admission to the neonatal intensive care unit (NICU), meconium-stained amniotic fluid, and neonatal Apgar scores at 1 and 5 min.

**Results:**

No differences were noted in the maternal age, body mass index (BMI) on admission, gestations, parity, gestational weeks at delivery and neonatal birth weight, or the rate of gestational diabetes mellitus (GDM) and hypertension disorder did not significantly differ among the three groups (*p* > 0.05). The no-NA group had shorter durations of first stage, second stage of labor compared to the early-NA or late-NA group (median, 215.0 min and 10.0 min vs. 300.0 min and 12.0 min vs. 280.0 min and 13.0 min) (*p* < 0.05), but no differences were observed between the early-NA and late-NA group (*p* > 0.05). There were no differences in the rate of intrapartum CD, intrapartum fever, PPH, maternal transferred to ICU, neonatal transfer to NICU, meconium-stained amniotic fluid, and postpartum stay ≥ 7d, as well as the neonatal the Apgar scores at 1 and 5 min among the three groups (*p* > 0.05).

**Conclusion:**

NA is associated with extended durations of the first, second and total stages of labor. However, the early initiation of NA in multiparous women (cervical dilation within 0.5-2.0 cm) does not increase the risk of intrapartum CD or intrapartum fever. These findings endorse the secure utilization of early NA for pain relief during labor in multiparous women.

## Introduction

Women’s preferences for pain relief during pregnancy vary significantly. Therefore, it’s crucial to facilitate discussions about pain management throughout their pregnancy journey [1]. Globally, neuraxial analgesia (NA) provides satisfactory pain relief during labor. However, its historical development has been linked to undesirable outcomes such as prolonged labor, an increased need for operative delivery, intrapartum cesarean delivery (CD), and intrapartum fever [2–4]. NA has been shown to prolong the first stage of labor by approximately 30 min and the second stage by 15 min compared to other forms of analgesia [2]. Initiating NA with lower cervical dilation and prolonged analgesia duration is associated with an increased risk of epidural-related maternal fever [5]. While pharmaceutical analgesia offers better pain relief and neonatal outcomes than nonpharmaceutical methods, it is associated with longer labor duration and increased postpartum bleeding [6]. High-quality studies have consistently found no increased risk of intrapartum CD and assisted vaginal delivery with NA, especially when using newer modalities such as low-concentration local anesthetic solutions equivalent to ≤ 0.1% bupivacaine, programmed intermittent epidural bolus, and patient-controlled NA [2]. The timing of NA initiation also influences outcomes for both mothers and neonates. Guesine GD et al. [7] reported that NA in parturients with cervical dilatation ≥ 9.0 cm increased the risk of forceps delivery by 3.86-fold and a higher prevalence of fetal bradycardia, a higher need for neonatal oxygen therapy, and a higher need for admission to a neonatal intensive care unit (NICU); NA in parturients with cervical dilatation ≤ 4.0 cm increased the risk of CD by 3.31-fold and a higher prevalence of Apgar score < 7 at 1 min.

Our study focused on investigating the effects of early (cervical dilation within 0.5-2.0 cm) and late (when the cervix was dilated ≥ 2 cm) initiation of NA in multiparous women who voluntarily underwent vaginal delivery on admission. We retrospectively assessed outcomes for both maternal and neonates, including labor progress, delivery mode, PPH, intrapartum fever, transfer to ICU, admission to NICU, meconium-stained amniotic fluid, and neonatal Apgar scores at 1 and 5 min, in comparison to those who did not receive NA. The initial dose administered of NA through the programmed intermittent epidural bolus (PIEB) pump consisted of 10mL of a drug mixture containing 0.08% ropivacaine and 2 µg/mL fentanyl [8].

## Materials & methods

### Ethical approval and patient consent

The study protocol received ethical approval from the Ethics Committee of Maternal and Child Health Hospital of Hubei Province ([2023] IEC (049)). Additionally, all participating women provided written informed consent for therapeutic procedures and for the publication of therapeutic procedures.

### Selection of patients and study design

**Fig. 1:**
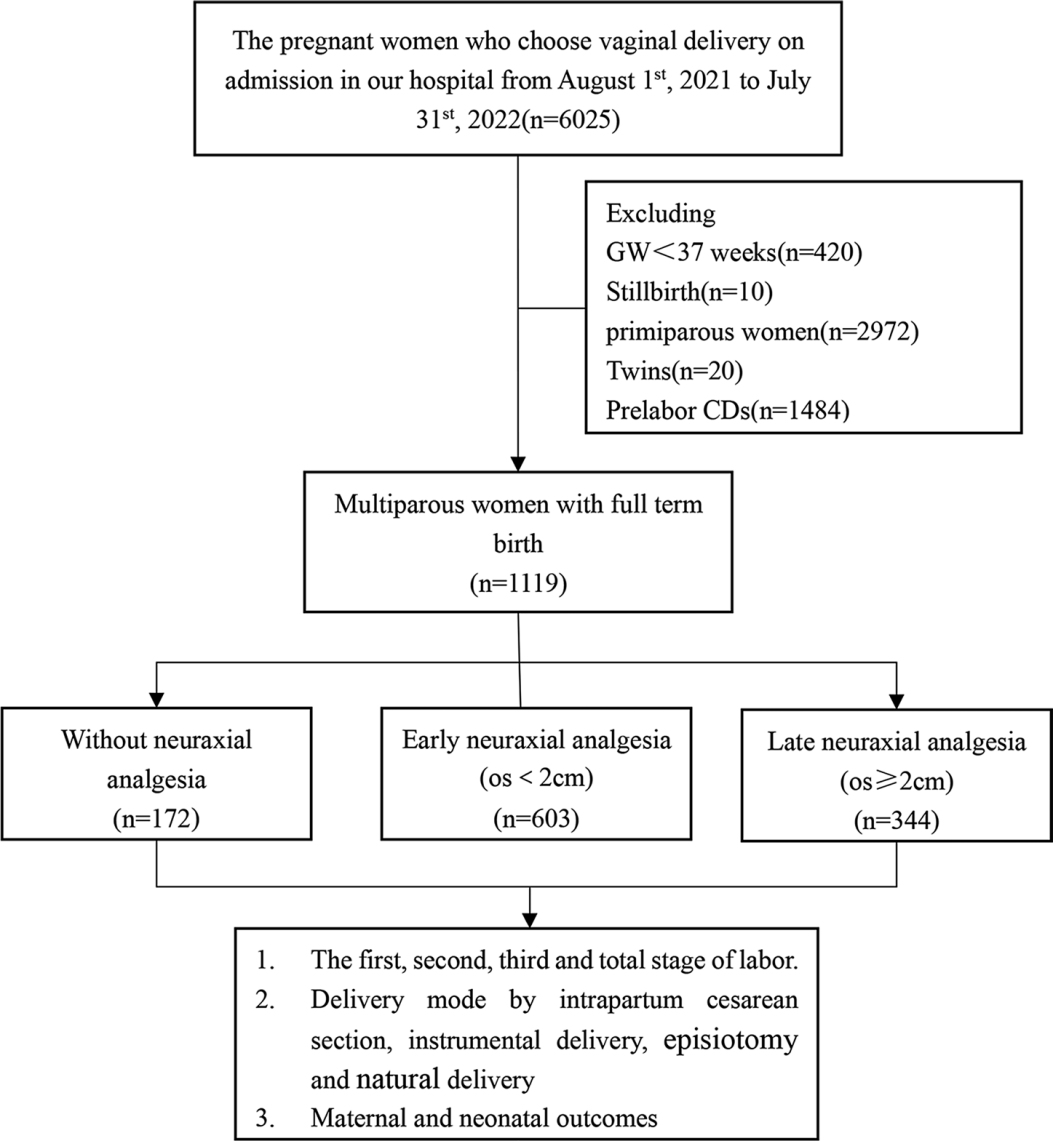
Study flowchart

The flowchart of the experimental design is depicted in Fig. 1. In this retrospective monocentric study, we enrolled pregnant women who were treated from August 1st, 2021 to July 31st, 2022 at our birth center (Optics Courtyard), a tertiary- level maternal and child health hospital in Hubei province, China. The inclusion criteria for our study were: (1) multiparous women; (2) cephalic presentation; (3) singleton pregnancy; (4) full-term births ranging from 37 + 0 weeks to 41 + 6 weeks; (5) live birth; (6) selection of vaginal delivery on admission. The exclusion criteria were as follows: (1) primiparous women; (2) premature delivery; (3) multiple pregnancies (e.g., twins); (4) stillbirth; (5) per-labor CD. In this study, a total of 6,025 pregnant women who initially chosen vaginal delivery on admission to our department were screened. From this initial cohort, we excluded 420 cases of premature delivery with gestational weeks less than 37 weeks, 10 cases of intrauterine fetal death, 2972 cases of primiparous women, 20 cases of twin pregnancies, and 1484 cases of elective CD. Ultimately, a total of 1,119 cases of multiparous women were included and recruited for further analysis.

Participants were categorized into three groups based on the choice of NA and the timing of NA initiation: no-NA, early-NA and late-NA. The no-NA group comprised 172 participants who did not receive NA during vaginal delivery. The early-NA group consisted of 603 participants who received NA when the cervix was dilated within 0.5-2.0 cm.The late-NA group included 344 participants who received NA when the cervix was dilated ≥ 2 cm.

### Methods of NA [8]

Multiparous women undergoing vaginal delivery with the willingness to use NA were assessed by both an anesthesiologist and obstetrician to evaluate their systemic situation and cervical conditions. NA was initiated by the anesthesiologist with the patient in the left lateral decubitus position, targeting the epidural space at the L3-4 or L4-5 interspace, following the administration of 500mL of Ringer’s lactate solution by an anesthesia nurse. A PIEB pump (Master PCA pump, Fresenius Kabi UAS, without continuous background infusion) was connected to the catheter. The initial dose administered through the pump consisted of 10mL of a drug mixture containing 0.08% ropivacaine and 2 µg/mL fentanyl. Subsequently, the pump automatically delivered 10mL/h through the PIEB pump at regular intervals. During labor, the patients were given the ability to self-administer additional pain relief by pressing a button on the PIEB pump whenever they felt discomfort. Each press of the button released a 10mL mixture of the drug, with the effective interval time for each press set at 15 min. The infusion was stopped after the stitching process was completed, and the catheter was typically removed within 2–3 h after delivery.

### Duration stage of labor [9]

The first stage of labor: encompasses the period from the onset of labor to full cervical dilation of 10 cm. Labor is typically defined as beginning when contractions become strong and occur regularly, usually around 3 to 5 min apart. The second stage of labor begins with complete cervical dilation of 10 cm and extends until the delivery of the baby. The third stage of labor begins immediately after the birth of the baby and continues until the delivery of the placenta.

### Intrapartum CD [10, 11]

The inclusion criteria for intrapartum CD included women with documented evidence of painful regular contractions or induction of labor prior to CD. Indications for CD encompassed non-reassuring fetal heart rate (NRFHR) or meconium-stained amniotic fluid (MSAF), labor dystocia (persistent occiput posterior/occiput transverse positions, etc.), failed induction of labor, intrapartum fever and other relevant factors (such as maternal request, placental abruption and umbilical cord prolapse).

### Intrapartum Fever [12]

Intrapartum fever was defined as tympanic temperature of 37.5 °C or higher and was found to be associated the administration of NA during labor. If the parturient developed intrapartum fever, prophylactic antibiotics were administered, and fluid replacement was accelerated to replenish the water lost as the tympanic temperature rose to 38^0^C-38.5^0^C. If the tympanic temperature rose above 38.5^0^C, oral administration of ibuprofen was initiated.

### Observation indicators

All data were obtained from electronic medical records. Demographic characteristics, including the maternal age, gestational week at delivery, body mass index (BMI) on admission, gestational times, parous times, neonatal birth weight, pregnancy complications such as Gestational Diabetes Mellitus (GDM) and hypertension disorder, were collected. Additionally, information on the first, second, third, and total stages of labor were recorded. Details regarding the methods of delivery, including intrapartum CD, operative vaginal delivery, natural delivery, and episiotomy, were also documented. Furthermore, maternal outcomes such as the amount and rate of postpartum hemorrhage (PPH), intrapartum fever, maximum temperature during labor, postpartum hospital stay duration, and the rate of transfer to the intensive care unit (ICU) were assessed. Neonatal outcomes, including Apgar scores at 1 and 5 min, the rate of admission to the neonatal intensive care unit (NICU), and the presence of meconium-stained amniotic fluid, were also evaluated.

### Sample size calculation

Sample size was calculated based on the duration of labor that we observed in this study. Assuming the median and standard deviation of duration of labor among three groups, α = 0.05 and β = 0.1, according to the following formula, 117 women without NA, 189 women who received NA when the cervix was dilated within 0.5-2.0 cm and 174 women who received NA when the cervix was dilated ≥ 2 cm were required to determine an effect.

$${n}_{ij}=\frac{{\left({Z}_{1?\alpha }/\left(2T\right)+{Z}_{1?\beta }\right)}^{2}\left({\sigma }_{1}^{2}+{\sigma }_{2}^{2}\right)}{{{\delta }_{ij}}^{2}}$$

$$\text{n}\hspace{0.17em}=\hspace{0.17em}\text{m}\text{a}\text{x}\left\{{n}_{ij},parirs\left(i, j\right)\right\}$$

### Statistical analysis

SPSS 28.0 was used for statistical analysis. If the measurement data were in line with normal distribution, they were presented as mean ± standard deviation $(\bar{x}\pm s)$. One-Way ANOVA and F test were performed to compare the variables in gaussian distribution. If the measurement data were in non-normal distribution, M(p25, p75) was used, and Kruskal-Wallis H test was used to assess the variables among three independent samples. Count data were expressed as frequencies or rates, Fisher’s exact test and χ^2^ test was used for comparison among the three groups. Statistical significance was set at a value of *p* < 0.05.

## Results

The baseline data for the no-NA, early-NA and the late-NA group are listed in Table 1. No significant differences were observed in the maternal age, BMI on admission, gestational times, parous times, gestational weeks on delivery, neonatal birth weight, the rate of GDM and hypertension disorder among the three groups (*p* > 0.05).

**Table 1:** Comparison of baseline parturients’ characteristics and comorbidities among the three groups

	No-NA group (n = 172)	Early-NA group (n = 603)	Late-NA group (n = 344)	F/χ^2^	*p* value
Maternal age (y, $\bar{x}\pm s$)	31.7 ± 3.6	32.1 ± 3.5	31.8 ± 3.3	1.577	0.207
BMI on admission (kg/m^2^, $\bar{x}\pm s$)	26.1 ± 2.9	26.5 ± 2.8	26.5 ± 2.8	1.803	0.165
Gestational times (n, $\bar{x}\pm s$)	3 ± 1.0	3 ± 1.0	3 ± 1.0	0.269	0.765
Parous times (n, $\bar{x}\pm s$)	2 ± 0.3	2 ± 0.3	2 ± 0.2	2.891	0.056
Gestational weeks (w, $\bar{x}\pm s$)	39.1 ± 0.9	39.2 ± 0.9	39.2 ± 0.9	1.801	0.166
Neonatal birth weight (g, $\bar{x}\pm s$)	3278 ± 317	3332 ± 362	3347 ± 372	2.210	0.110
GDM (n, %)	35, 20.3	125, 20.7	69, 20.1	0.062	0.969
Hypertension disorder (n, %)	8, 4.7	41, 6.8	28, 8.1	2.191	0.334

NA, neuraxial analgesia; GDM, Gestational Diabetes Mellitus; BMI, body mass index

One-way ANOVA, F test and χ^2^ test were used

The median durations of the first, second and total labor periods in the no-NA, early-NA, and late-NA groups were 215.0 min, 10.0 min, 230.0 min vs. 300.0 min, 12.0 min, 325.0 min vs. 280.0 min, 13.0 min, 304.0 min respectively. These durations were significantly shortest in the no-NA group (*p* < 0.05), but these were no difference between in the early-NA and late-NA group (*p* > 0.05). The average extension time of the third stage of labor showed no significant difference among the three groups (*p* > 0.05). Data are displayed (Table 2).

**Table 2:** Comparison of labor progress among the three groups

	No-NA group (n = 165)	Early-NA group (n = 592)	Late-NA group (n = 340)	H	*p* value
Duration of 1st stage of labour [min, Median(p25, p75)]	215.0 (160.0, 290.0)	300.0^*^ (220.0, 420.0)	280.0^*^ (210.0, 390.0)	61.422	0.000
Duration of 2nd stage of labour [min, Median(p25, p75)]	10.0 (7.0, 13.0)	12.0^*^ (9.0, 18.0)	13.0^*^ (8.0, 18.0)	37.020	0.000
Duration of 3rd stage of labour [min, Median(p25, p75)]	7.0 (5.0, 10.0)	6.0 (5.0, 9.0)	6.5 (5.0, 9.0)	5.105	0.078
Total duration of labour [min, Median(p25, p75)]	230.0 (175.0, 315.0)	325.0^*^ (240.0, 445.0)	304.0^*^ (235.0, 411.0)	63.238	0.000

NA, neuraxial analgesia

Kruskal-Wallis H test were used

*Compared with the no-NA group, *p*<0.05

For delivery methods, the data indicated that the rate of intrapartum CD was 4.1% (7/172) in the no-NA group, 1.8% (11/603) in the early-NA group, and 1.2% (4/344) in the late-NA group. No significant differences were observed among the three groups. (*p* > 0.05). The rates of naturally vaginal delivery, episiotomy, and forceps delivery also showed no difference among the three groups (*p* > 0.05). Data are displayed (Table 3).

**Table 3:** Comparison of delivery mode among the three groups

	No-NA group (n = 172)	Early-NA group (n = 603)	Late-NA group (n = 344)	χ^2^/R	*p* value
Natural delivery (n, %)	161, 93.6	572, 94.9	327, 95.1	0.530	0.767
Episiotomy (n, %)	3, 1.7	18, 3.0	12, 3.5	1.225	0.542
Forceps delivery (n, %)	1, 0.6	2, 0.3	1, 0.3	0.830	0.808
Intrapartum CD (n, %)	7, 4.1	11, 1.8	4, 1.2	4.628	0.099

NA, neuraxial analgesia;CD, cesarean delivery

Fisher’s exact test and χ^2^ test were used

The maternal outcomes were analyzed among the three groups, encompassing parameters such as the amount of blood loss, maximum intrapartum temperature, the rate of PPH and intrapartum fever, postpartum stay ≥ 7 days and admission to ICU. No significant differences were observed among the three groups for these outcomes. (*p* > 0.05). Data are displayed (Table 4).

**Table 4:** Comparison of maternal outcomes among the three groups

	No-NA group (n = 172)	Early-NA group (n = 603)	Late-NA group (n = 344)	F/χ^2^/R	*p* value
The amount of blood loss (mL, $\bar{x}\pm s$)	300 ± 113	314 ± 108	314 ± 105	1.266	0.282
PPH (n, %)	6, 3.5	34, 5.6	16, 4.7	1.432	0.489
Intrapartum fever (n, %)	5, 2.9	17, 2.8	10, 2.9	0.008	0.996
Maximum temperature (℃, $\bar{x}\pm s$)	36.8 ± 0.3	36.8 ± 0.3	36.8 ± 0.3	0.626	0.535
PP stay ≥ 7d (n, %)	3, 1.7	9, 1.5	5, 1.5	0.241	0.945
Admission to ICU (n, %)	4, 2.3	12, 2.0	7, 2.0	0.225	0.957

NA, neuraxial analgesia; PPH, postpartum hemorrhage; PP, postpartum; ICU, intensive care unit

One-way ANOVA, F test, Fisher’s exact test and χ^2^ test were used

There was no difference among the three groups in the Apgar score at 1 and 5 min, the rate of new babies transferred to NICU and the incidence of meconium-stained amniotic fluid (*p* > 0.05). Data are displayed (Table 5).

**Table 5:** Comparison of neonatal outcomes among the three groups

	No-NA group (n = 172)	Early-NA group (n = 603)	Late-NA group (n = 344)	H/χ^2^	*p* value
Apgar score at 1 min (point, $\bar{x}\pm s$)	9.9 ± 0.3	9.9 ± 0.4	9.9 ± 0.4	1.659	0.436
Apgar score at 5 min (point, $\bar{x}\pm s$)	10.0 ± 0.2	10.0 ± 0.1	10.0 ± 0.1	0.499	0.779
Admission to NICU (n, %)	6, 3.5	17, 2.8	12, 3.5	0.411	0.814
Meconium-stained amniotic fluid (n, %)	12, 7.0	40, 6.6	30, 8.7	1.442	0.486

NA, neuraxial analgesia; PPH, postpartum hemorrhage; PP, postpartum; ICU, intensive care unit

Kruskal-Wallis H test and χ^2^ test were used

## Discussion

This study demonstrated that both early-(cervical dilation within 0.5-2.0 cm) and late-NA (cervical dilation ≥ 2.0 cm) groups compared with no-NA group were associated with a prolonged duration of the first, second and total stages of labor. However, there was no significant difference in labor progress between the early- and late-NA groups. Additionally, there were no difference in intrapartum CD or intrapartum fever among the early-NA, late-NA and no-NA groups.

NA is widely acknowledged as the most effective method of pain relief during labor in numerous healthcare settings. Laboring women often require the quicker onset provided by the dural puncture epidural technique and early-NA to alleviate pain during cervical dilation of less than 2-3 cm [13, 14]. At the same time, NA is associated with a prolonged duration of first, second and total stage of labor [2, 15, 16], intrapartum CD, operative vaginal delivery [15], intrapartum fever [3, 4], and adverse maternal and neonatal outcomes [17]. With the implementation of the three-child policy, there has been an increase in multiparous women opting for vaginal delivery in China. Early -NA in multiparous women was observed in our study.

Numerous studies consistently demonstrated that NA prolongs the durations of the first, second, and total stages of labor [2, 15, 16]. It has been suggested that the use of NA may prolong the duration of the first stage of labor by around 30 min and the second stage by approximately 15 min compared to labor without NA [2]. Shen X et al. found that maintaining the infusion of epidural medication had no significant effect on the duration of the second stage of labor [18]. In elective induction of multiparous women, the duration of the first and second stage of labor did not differ significantly between the early (NA was initiated when the cervical dilation was ≤ 3 cm) and late group (NA was initiated when the cervical dilation was > 3 cm) (median: 232 min, 37 min vs. 260 min, 40 min) [14]. Our findings were consistent with those studies. In our study, the median durations of the first, second, and total labor stages in the no-NA group were 215.0 min, 10.0 and 230.0 min, respectively, which were shorter than those in the early-NA group (300.0 min, 12.0 and 325.0 min) and late-NA group (280.0 min, 13.0 and 304.0 min). However, no difference was found between early-NA and late-NA multiparous women. The average extension time of the third stage showed no significant difference among multiparous women in the no-NA, early-NA and late-NA groups.

Numerous studies have consistently reported a significant increase in the incidence of intrapartum fever among parturients who received NA [3, 4, 19]. Intrapartum fever was associated with intrapartum CD, operative vaginal delivery, and PHH. Various high-risk factors have been identified for intrapartum fever, including higher maternal BMI on delivery, nulliparity, increasing gestational age, longer duration of labor, premature rupture of membranes, an increasing number of vaginal examinations, oxytocin use, higher birth weight, lower cervical dilation at the initiation of NA, and longer analgesia duration [5]. In our study, the rate of intrapartum fever and max intrapartum temperature showed no difference among the three groups. The lack of difference may be attributed to the specific focus of our study on multiparous women. The labor progress in multiparous women is generally faster than that in primiparous women. The rate of intrapartum fever was low among the no-NA, early- NA and late-NA groups (2.9%, 2.8% and 2.9%).

Numerous studies suggests that NA is associated with higher rates of intrapartum CD and operative vaginal deliveries [3, 15, 16]. Some studies [15, 20] found that NA resulted in a lower rate of episiotomies in primiparous women, but was associated with a higher rate of instrumental vaginal deliveries and intrapartum CD. Yagi T et al [20] observed a higher CD rate in deliveries with NA compared to those without NA in Robson group 1 pregnancies and Robson group 2a pregnancies. Among multiparous women, there was no significant difference in the rates of instrumental delivery and intrapartum CD between early-NA(initiated at cervical dilation was ≤ 3 cm) and late-NA(initiated at cervical dilation was ≤3 cm) [14]. Wong CA et al. also reported that NA in early labor (when the cervix is less than 4.0 cm dilated) did not increase the rate of CD [21]. Deepak D et al. also found that NA did not increase the risk of CD and instrumental vaginal birth [22]. Our study similarly revealed no significant differences in the rates of intrapartum CD, forceps-assisted vaginal delivery, episiotomy and natural vaginal delivery among multiparous women in the no-NA, early-NA and late-NA groups.

The influence of NA on maternal and neonatal outcomes has been extensively investigated and continues to be a subject of ongoing research [2, 16, 23]. Guglielminotti J et al. [24] found that the use of NA is associated with a 14% decrease in the risk of severe maternal morbidity, with more than one-fifth of the observed association attributed to the decreased risk of PPH. These findings suggest that the utilization of NA has the potential to improve maternal health outcomes. For women receiving neuraxial analgesia, both primipara and multipara, there was a notable increase in PPH and a decrease in umbilical base excess values [25]. In multiparas, combined spinal-epidural analgesia (CSEA) provided superior analgesia and satisfaction compared to remifentanil patient-controlled analgesia (RPCA) [26]. After propensity score matching, Watanabe K and colleagues discovered that the nulliparous NA group exhibited a notably higher incidence of Apgar scores of 7 at both 1 and 5 min and had increased occurrences of meconium-stained amniotic fluid, while no significant difference in neonatal outcomes were observed in multiparous women [27]. Our study found that there was no significant difference in the amount and rate of PPH, the rate of transferred to ICU, postpartum stay ≥ 7d, the Apgar score at 1 and 5 min, the rate of neonatal transfer to NICU and the rate of meconium-stained amniotic fluid among the no-NA, early-NA and the late-NA multiparous women. This indicates that initiating NA early during labor in multiparous women can effectively address the demand for pain relief while maintaining the safety of both maternal and newborns.

### Strengths and limitations

Our study has the advantage of being conducted in a single-center setting with uniform practices in anesthesia administration, drug concentration, and NA methods. This uniformity enhances the reliability of our findings concerning the relationships among no-NA, early-NA, and late-NA multiparous women regarding maternal and neonatal outcomes. Additionally, our study specifically focuses on early-NA, a highly desired pain relief method for laboring women, and includes data from multiparous women who did not receive NA.

However, our study has certain limitations. It is retrospective and observational, conducted at a single-center, which may limit the generalizability of our findings. To provide a more comprehensive understanding, future research should involve multicenter studies that encompass all stages of labor for women who choose early-NA.

## Conclusion

This study revealed that, when compared to late-NA, early-NA for multiparous women in China is both safe and effective. It is noteworthy that the use of early-NA did not lead to an increase in the rate of intrapartum CD, longer duration of labor stages, or any adverse effects on maternal and neonatal outcomes.

## Data Availability

All of the data are included in the article. Further inquiries may be sought from the corresponding author upon reasonable request.

## Abbreviations

NA: neuraxial analgesia
GDM: Gestational Diabetes Mellitus
BMI: body mass index
PPH: postpartum hemorrhage
CD: cesarean delivery
NRFHR: non-reassuring fetal heart rate
MSAF: meconium-stained amniotic fluid
PP: postpartum
ICU: intensive care unit
NICU: neonatal intensive care unit
CSEA: combined spinal-epidural analgesia
RPCA: remifentanil patient-controlled analgesia
